# Concurrent locomotor adaptation and retention to visual and split-belt perturbations

**DOI:** 10.1371/journal.pone.0279585

**Published:** 2022-12-30

**Authors:** Seung-Jae Kim, Scott Howsden, Nicole Bartels, Hyunglae Lee

**Affiliations:** 1 Biomedical Engineering, California Baptist University, Riverside, California, United States of America; 2 School for Engineering of Matter, Transport, and Energy, Arizona State University, Tempe, Arizona, United States of America; Kennedy Krieger Institute/Johns Hopkins University School of Medicine, UNITED STATES

## Abstract

Gait asymmetry is a common symptom in groups with neurological disorders and significantly reduces gait efficiency. To develop efficient training for gait rehabilitation, we propose a novel gait rehabilitation paradigm that combines two distinct perturbation strategies: visual feedback distortion (VFD) and split-belt treadmill (SBT) walking. In SBT walking, spatiotemporal gait adaptation can be readily achieved, but it quickly fades after training. Gait adaptation to implicit VFD in an unconscious manner tends to persist longer, potentially due to a greater engagement of implicit learning during training. Thus, we investigated whether the combined strategies would lead to more effective changes in symmetric gait patterns with longer retention periods. We compared the retention of the preserved asymmetric pattern acquired by “implicit VFD+SBT walking” with “SBT-only walking” and with “SBT walking with conscious correction”. In the implicit VFD+SBT walking, the speed of the two belts was gradually changed, the visual representation of gait symmetry was implicitly distorted, and no instructions were given to subjects except to watch the visual feedback. In the SBT walking with conscious correction, subjects were instructed to consciously correct their steps with the help of visual feedback while SBT walking. The SBT-only walking consisted of SBT walking with no visual feedback. After the 7-minute adaptation period, we removed the visual feedback and the split-belt perturbations, and we assessed the retention of the preserved asymmetric pattern while subjects continued walking for the 15-minute post-adaptation period. In a group of subjects who spontaneously showed visuomotor adaptation in response to the implicit VFD (16 out of 27 subjects), we found a greater retention rate during the implicit VFD+SBT walking trial than the SBT-only walking or the SBT walking with conscious correction trials. The implicit visual distortion paradigm delivered in an attention-independent (unconscious) manner can be utilized and integrated into SBT walking to improve the efficacy of symmetric gait adaptation by producing longer-lasting effects on the retention of a newly learned motor pattern.

## Introduction

Gait asymmetry is one of the most common conditions following neurological impairments, such as a stroke, Parkinson’s disease (PD), or multiple sclerosis (MS) [1–3]. Deficits in temporal and spatial gait symmetry produce gait abnormalities, such as reductions in walking speed and balance [4, 5] and unbalanced propulsions between the legs [6]. Thus, restoring spatiotemporal gait symmetry is an important goal in gait rehabilitation to minimize challenges that face many neurologic populations.

In an effort to alleviate spatial asymmetric gait, split-belt treadmill (SBT) walking training has been tested [7, 8], where the different belt speeds exaggerate patients’ step length asymmetry. For instance, when the different split-belt speeds exaggerate stroke patients’ step length asymmetry, they start to adapt their gait pattern by increasing their shorter step length on the faster belt. When the two belts are returned to the same speed, the gait adaptation to SBT walking has been shown to produce aftereffects of newly adapted symmetric gait patterns, which can lead to temporal corrections in spatiotemporal gait symmetries [9–13]. However, the aftereffects in response to the mechanical perturbations by SBT are short-lived (i.e., poor retention of the adapted gait patterns) [14–16], which results in a limited transfer of newly learned motor patterns to post-training movements [17].

In motor learning, the interaction between explicit and implicit learning processes is related to consolidating motor memories [18], and there has been considerable interest in the role of implicit processes in motor adaptation and retention [19–21]. An implicit learning process generally refers to acquiring a certain motor pattern without voluntary or conscious movements. As an alternative training intervention to modulate gait symmetry in a non-conscious manner, we have previously proposed a novel visual perturbation paradigm in which subjects adapt their gait patterns in response to visual feedback distortion (VFD) of their step length symmetry [22, 23]. For this, we distorted the visual feedback of subjects’ gait step symmetry so that subjects perceived their gait asymmetrically. The subjects were not informed that the feedback was being distorted, and they were not given any experimental tasks where they had to consciously perform an action during the trials. We found that a gradual distortion of visual feedback systematically modulated gait step length away from symmetry and the adapted gait pattern (aftereffects) remained even after the visual feedback was removed [17]. The subjects did not notice distortion or modulation of their symmetric gait pattern. In addition, our prior study on healthy subjects compared the magnitude of aftereffects of step length asymmetry acquired using implicit VFD versus SBT walking [15], and we discovered that subjects who were trained with VFD retained aftereffects significantly longer compared to the SBT walking trial. These results suggest that distorted visual information in an unconscious manner can create sensory-prediction errors and provide a greater degree of implicit motor memory, which is thought to be responsible for the longer retention of newly learned motor patterns [9, 17, 19, 20].

SBT walking is a well-characterized gait learning intervention via mechanical perturbation and can induce sizeable changes in spatiotemporal gait symmetry. One way to improve the efficacy of symmetric gait adaptation and retention would be to integrate the visual distortion and the mechanical perturbations into a single training session. Gait control may employ distinct adaptive learning processes during walking under distinct perturbations, and the different perturbation modalities could potentially have complementary effects on gait symmetry adaptation and retention. For visual perturbations, while walking on a split-belt treadmill, visual bars that represent the right and the left step lengths are displayed on a computer screen, and only one of the bars is distorted without subjects’ knowledge of the distortion. The mismatch between the predicted and actual visual information of gait symmetry may induce asymmetric gait patterns with a longer retention effect [22, 23]. Thus, we aimed to investigate the potential benefits of concurrent gait training that combined VFD with SBT walking in healthy individuals. We evaluated the short-term retention (aftereffects) of adapted spatial gait symmetry from the implicit VFD+SBT combining strategy and compared them to those with SBT-only walking. We hypothesized that the concurrent gait adaptation to implicit VFD+SBT perturbations in an unconscious manner produces complementary benefits of facilitating the adaptation process and retaining adapted gait patterns longer than the single mechanical perturbation strategy [12]. We also compared the retention from the implicit VFD+SBT walking trials with the SBT walking with conscious correction trials to confirm that unconscious processes associated with motor adaptation can have longer-lasting retention effects than conscious (explicit) processes. A positive outcome of this study would indicate that implicit visual perturbations through VFD can be useful for gait rehabilitation when used in conjunction with SBT walking because motor learning has a better retraining effect.

## Materials and methods

### A. Subjects

Thirty healthy adult volunteers, 20–50 years with no visual or physical disability, participated in this study. The subjects’ average age was 22.6 ± 6.2 years, their average weight was 71.5 ± 12.1 Kgs, and their average height was 174.7 ± 9.5 cm. Subjects were given a detailed explanation of the study procedure by the researchers before providing their written informed consent to participate in this study. All subjects were informed of the physical fatigue they would experience during the experiment and were accustomed to walking on a treadmill. All protocols (14-EF-023) were approved by the Institutional Review Board of California Baptist University to confirm the study meets national and international guidelines for research on humans.

### B. Experimental setup

All walking experiments were performed using a split-belt treadmill (Woodway USA, Waukesha, WI) consisting of two separate belts, each with its own motor that permitted the speed of each belt to be controlled independently. The treadmill was equipped with supporting handrails to ensure the safety of subjects. All subjects were given a 5–10 minute habituation period to adjust to walking on the treadmill. Two motion-capturing markers were attached to the back of the subject’s shoes. These markers were seen by an optometric motion capture system (OPTOTRACK 3D Investigator, Northern Digital Inc., Canada) that locates the positions of the markers. The retrieved data was then sent to a PC in real-time using a program developed with LabVIEW (National Instruments Corp., TX) to graphically represent the subjects’ step length measurements on a computer screen.

**Fig 1:**
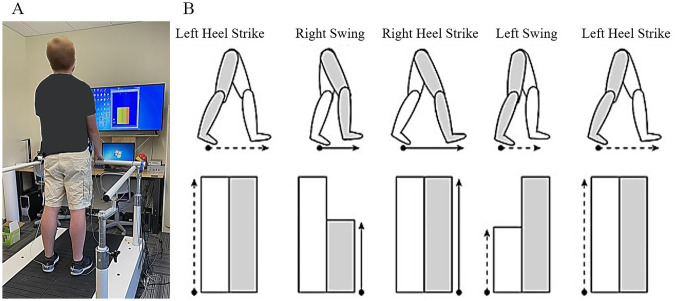
Experimental setup showing an instrumented treadmill and visual feedback. **A**: Three infrared cameras were placed behind to capture the two markers attached to the subjects’ shoes. **B**: Visual representation of subjects’ movement was captured using a motion capture system. The range of the right and left step lengths was mapped to the vertical bars. During the swing phase of a leg, the corresponding bar increases proportional to the step length and stops when a heel strike occurs for that leg. For visual distortion, the range of right step length mapped to the visual bar was gradually distorted.

For visual feedback, real-time visual feedback of step length was provided to subjects on a 48-inch LCD display placed directly in front of the treadmill (about 2 meters away). The display shows two vertical bar graphs representing the step length of each leg (Fig 1). The step length is defined as the distance between the two legs, expressed as the vertical bar’s height. Thus, during the swing phase of a leg, the height of the corresponding vertical bar initially had no height and increased in real time. The height of the vertical bar freezes when a heel strike occurs, and it resumes to change when that leg reenters the next swing phase and passes the contralateral stance foot (i.e., when the distance is zero). Therefore, subjects observe the maximum stride length of the right and the left leg side by side at every heel strike. We thoroughly explained the meaning of the bar to the subjects.

During the visual feedback distortion trial, we distorted the length of only the right bar (representing the right step length) in 8% decrements from 100% down to 60%. For example, a -8% distortion of step length changed the bar height from 100% to 92%. Thus, subjects would perceive their right step length to be 8% shorter than the actual length during the visual distortion. Each distortion level lasted for 60 seconds for the first 7-minute adaptation period. After the adaptation phase, the visual feedback was removed, and the subjects continued walking for the remaining 15-minute post-adaptation period. Our post-test questionnaire confirmed that the subjects did not notice the distortion.

### C. Experimental protocol

The experiments were designed a) to primarily investigate the effects of visual feedback distortion within an unconscious manner on the retention of the new gait pattern (asymmetric gait pattern) with the SBT walking trial and b) to see if there was an advantage of using the implicit visual distortion strategy versus the explicit conscious correction strategy in terms of the retention of the newly learned gait pattern. 30 subjects participated in two different trials at least several days apart: 1) a trial with SBT walking only (***SBT-only***) and 2) a trial with SBT walking combined with implicit visual feedback distortion (***implicit VFD+SBT***). Of the 30 subjects, 12 subjects also participated in a conscious correction trial that combined SBT walking with veridical visual feedback (***conscious VF+SBT***). In this additional trial, the subjects consciously (voluntarily) corrected their asymmetric gait pattern under SBT walking perturbation. Due to the mechanical perturbation by SBT, the two vertical bars would look asymmetric, and the subjects were then asked to match the height of the bars over the entire adaptation period. The order of trials was randomized between the SBT-only and combined trials. As for the combined trials, the implicit VFD+SBT trial was performed at intervals of several days before the conscious VF+SBT trial. Table 1 summarizes the three experimental conditions tested in this study.

**Table 1:** Summary of experiments.

Experimental Condition	Description
SBT-only	Subjects walked on a split-belt treadmill with no visual feedback of gait symmetry. The split-belt perturbation was removed (tied-belt) during the post-adaptation period.
Implicit VFD+SBT	Subjects walked on a split-belt treadmill with distorted visual feedback of gait symmetry. The subjects were not informed that the visual feedback was distorted, and no instruction was given except to walk comfortably (non-conscious learning). The split-belt perturbation and visual feedback were removed during the post-adaptation period.
Conscious VF+SBT	Subjects walked on a split-belt treadmill with veridical visual feedback of gait symmetry. They were instructed to match visual bars by correcting their asymmetric steps (conscious learning). The split-belt perturbation and visual feedback were removed during the post-adaptation period.

Each experimental trial consisted of a 5-minute control and 25-minute main sessions. For the first control trial, the subjects walked comfortably while the treadmill belts were moving at the speed of 2 mph (0.894 m/s), whereas during the control trial on their second visit, they were asked to look at visual feedback (no distortion applied) on the screen while walking. For the 25-minute main trial, there were three distinct sections: the baseline period (3 minutes), the adaptation period (7 minutes), and the post-adaptation period (15 minutes). The treadmill’s speed during the pre-adaptation phase was a consistent speed of 2 mph for the 3 minutes. During the adaptation period for all three conditions (*SBT-only*, *implicit VFD+SBT*, and *conscious VF+SBT*), the speed of the left leg stayed at 2 mph while the right leg speed was increased from 2 to 3 mph (1.341 m/s) by 0.2 mph (0.0894 m/s) every minute. The speed of the right belt reached 3 mph after 4 minutes and stayed at 3 mph for another 3 minutes until the post-adaptation phase started. During the 15-minute post-adaptation period, the speeds of the two belts returned to the same speed (2 mph), and the visual feedback was also removed. The subjects continued to walk until the end of the trial. During the experimental session, we measured the changes in gait symmetry and assessed the retention of asymmetric gait patterns during the post-adaptation period.

For the *implicit VFD+SBT* trial, the split-belt speed ratio change was identical to the *SBT-only* trial. Subjects were asked to keep their gaze on the screen and walk comfortably with no further specific instructions. For the initial pre-adaptation period, visual feedback without any distortion was shown to the subjects. During the adaptation period, only the right step length’s visual feedback was distorted by -8% every minute until a total of 60% distortion was achieved (in this way, visual feedback in the implicit VFD+SBT condition was distorted in a manner to exaggerate the typical step length asymmetry by SBT walking). Following the adaptation period, the visual feedback disappeared from the screen, the speeds of the two belts returned to the same speed, and subjects continued to walk for the remaining 15 minutes of post-adaptation. This was considered an implicit condition because the subjects were given visual feedback without knowing that the visual feedback of their step length was being distorted, and there was no experimental task they had to perform consciously during trials. They also did not notice the asymmetric step pattern caused by the visual distortion. In the *conscious VF+SBT* trial, the split-belt speed ratio change was identical to the *SBT-only* trial. However, subjects were asked to consciously match the heights of the visual feedback bars to induce greater asymmetric adaptation during the adaptation period.

### D. Data analysis

The primary measure used in this study was step length symmetry. To compute the step length symmetry, the ratio (%) between the left step length and the right step length was calculated for each gait cycle by using the following formula: 100×(right step length−left step length)/(0.5×(right step length + left step length)). Positive symmetry ratios mean that the right step lengths are longer than the left ones, and negative symmetry ratios indicate that the right step lengths are shorter than the left ones. Among the 30 subjects, three did not exhibit typical changes in step length symmetry in the implicit VFD+SBT walking. This was most likely because they tried to voluntarily change their gait steps by matching the heights of the two visual bars, even though it was an implicit (unconscious) condition. Thus, these three subjects’ data were excluded from the data analysis. To analyze the change in step length symmetry during the adaptation period (7 minutes) and the post-adaptation period (15 minutes) over time, the step length symmetries of each step over 30-second intervals were averaged. Then, the group means and SDs of the step length symmetry were calculated across all the subjects. In our experiments, healthy subjects showed some variation (2.6% on average) in step length symmetry ratio during the baseline walking. Thus, to minimize the variability of gait symmetry among subjects, the baseline step length symmetry (the symmetry value measured in the last two minutes of the baseline period) was subtracted from step length symmetry data.

The main goal of the present study was to compare the amount of adaptation and short-term retention (aftereffects) between the *implicit VFD+SBT* trial and the *SBT-only* trial, as well as between the *conscious VF+SBT* trial and the *SBT-only trial*. We did statistical analysis for the 7-minute and 15-minute post-adaptation periods separately. Two-way repeated measures ANOVA was performed to see if different experimental trials had a significant effect on the changes in step length symmetry. The two factors used for this analysis were the experimental conditions (different training strategies) and the level of the split-belt ratio or the visual distortion (for the analysis during the adaptation period) or the time (for the analysis during the post-adaptation period). In these analyses, Mauchly’s test of sphericity was used to test the assumption of sphericity, and the degrees of freedom were adjusted accordingly using the Greenhouse-Geisser correction before calculating the *p*-value. All statistical tests were performed at a significance level of *p* < 0.05. The *p*-values are displayed in the main text unless depicted in Figs. Additionally, we performed a paired t-test to examine at which level of perturbation (or at which time) the two training strategies showed a significant difference in step length symmetry.

## Results

**Fig 2:**
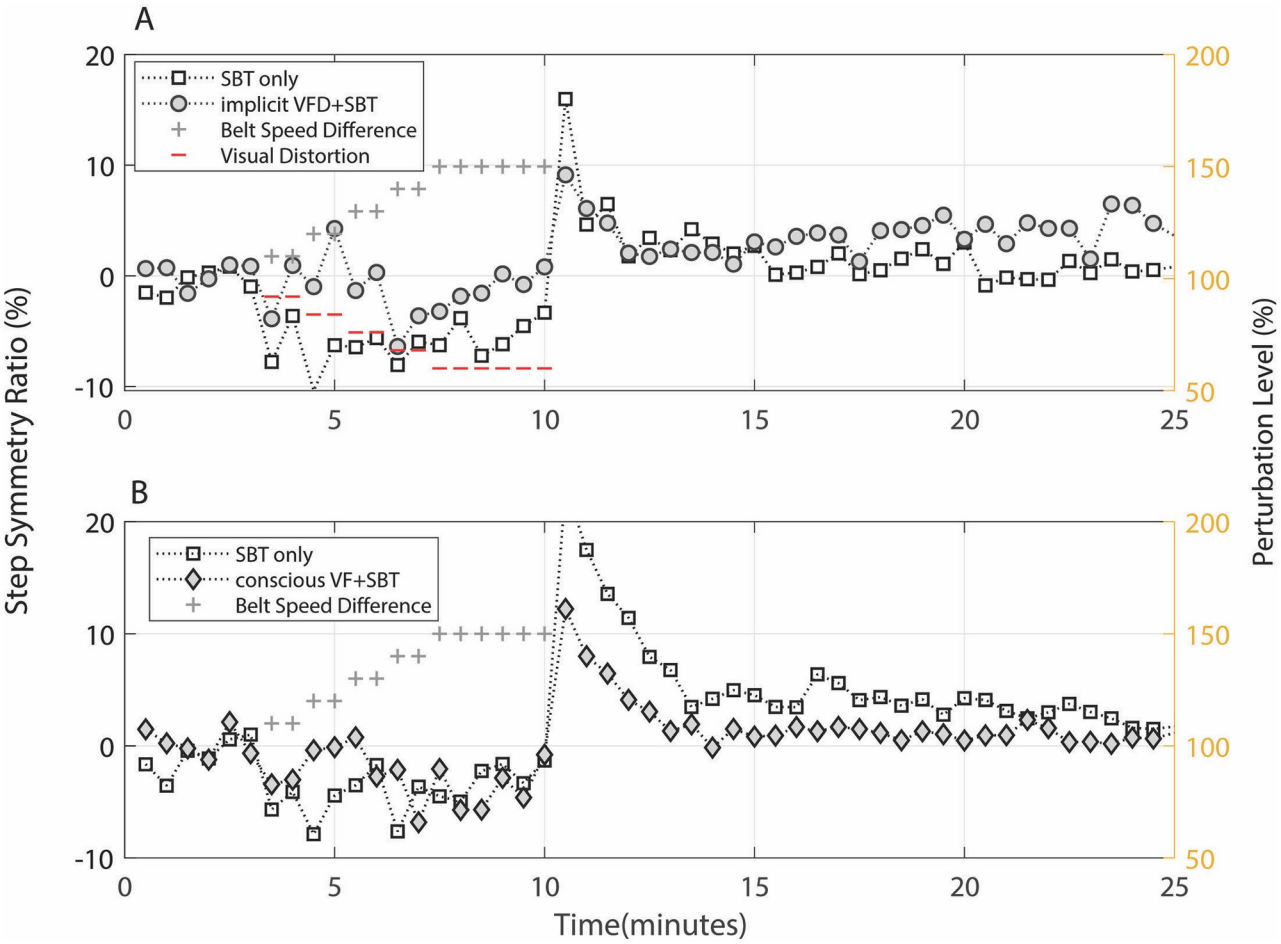
Changes in step length symmetry. **A**: Example of changes in step length symmetry resulted from the SBT-only trial and the implicit VFD+SBT trial obtained from a subject. **B**: Example of changes in step length symmetry resulted from the SBT-only and the conscious VF+SBT trials obtained from another subject. In all trials, visual distortion and split-bet perturbations were only used during the first 13 minutes (adaptation period). The intermittent horizontal lines indicate distortion or perturbation increments applied during those time periods. The horizontal axis shows time; the vertical axis shows the step length symmetry ratio between the actual left and right step lengths. The markers represent the mean step length symmetry value averaged over 30-second intervals at a given time.

Fig 2A shows an example of changes in step length symmetry obtained from a subject as a function of time for the implicit VFD+SBT and SBT-only trials. Each point represents the mean step length symmetry (%) of strides averaged over 30-second intervals. We observed a downward trend in the step length symmetry in the 7-minute adaptation period following the first 3-minute baseline period. If the speed ratio of the two belts had not been gradually adjusted, the subject’s step length symmetry would have approached the baseline (zero step length symmetry ratio) over time [10, 19]. However, in our experiment, the subject showed asymmetric gait throughout the adaptation period, as the two-belt speed ratio increased every minute. In the implicit VFD+SBT trial, the visual bar of the right step length was implicitly distorted shorter than the actual step length (the subject was unaware of the manipulation), and we observed reduced asymmetric gait patterns (closer to the baseline symmetry) during the adaptation period compared to the SBT-only trial. This indicates that the subject showed visuomotor adaptation (implicitly adapted to the asymmetric visual information) by producing longer right strides than during the SBT-only trial. During the following 15-minute post-adaptation period, the subject showed aftereffects of the adapted asymmetric pattern immediately after the speed of the two belts became the same, and the step length symmetry steadily decreased (moved toward the baseline). However, the rate of de-adaptation appeared to be different between the two trials. While the step length symmetry rapidly dropped towards the baseline in the SBT-only trial, the implicit VFD-SBT trial seemed to retain the asymmetric pattern longer.

Fig 2B shows an example of changes in step length symmetry obtained from a different subject for the conscious VF+SBT and SBT-only trials. During the adaptation period, the subject was told to match the height of the visual bars. If the subject had matched the bar perfectly, the symmetry ratio would have been very close to the baseline (zero), which was not the case for this subject. We observed that the step length symmetry rapidly decreased towards the baseline during the post-adaptation period in both trials.

**Fig 3:**
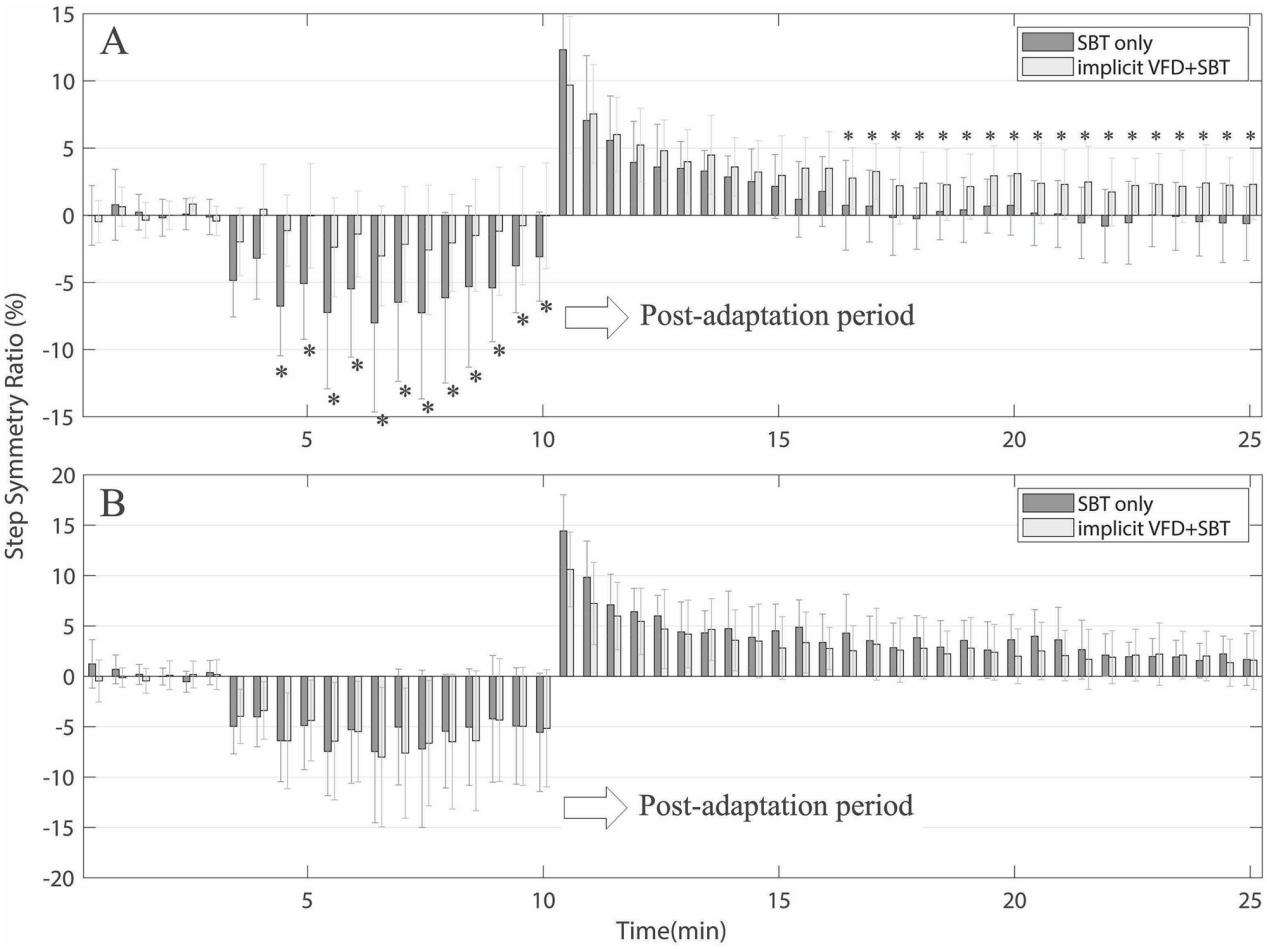
Group results of averages step length symmetry over time. **A**: Changes in the mean step length symmetry due to varying visual distortion and split-belt perturbation averaged across 16 subjects who showed visuomotor adaptation during the implicit VFD+SBT and SBT-only conditions. **B**: Changes in the mean step length symmetry of 11 subjects who did not show visuomotor adaptation for the same conditions. The error bars indicate the standard deviation of a reported mean step length symmetry for each level/time. The asterisks (*) were marked at time periods, where the induced step length symmetry values were significantly different between different adaptation conditions (p<0.05).

According to the data, we found only 16 subjects (adapted group) who showed visuomotor adaptation in response to implicit VFD while walking on a split-belt treadmill and 11 subjects (non-adapted group) who did not show visuomotor adaptation. The adapted group showed reduced gait asymmetry than that measured in the SBT-only trial during the perturbation period. On the other hand, the non-adapted group was clearly different from the adapted group in that their gait symmetry changes were similar to the typical changes shown in the SBT-only trial. Fig 3A shows the group means and variabilities in step length symmetry during the SBT-only trial versus the implicit VFD+SBT trial from the adapted group (16 subjects). The trend shown in Fig 2A was consistently observed in the group results (Fig 3A). We first observed the extent of how the two different groups changed their step length symmetry over the course of the adaption period. Statistical analysis using two–way repeated measures ANOVA on the step length symmetry revealed that there was a significant effect of trials on the changes in step length symmetry during the adaptation period (*F(1*, *15) = 45*.*758 with the Greenhouse-Geisser correction*, *p < 0*.*001*, *partial η*^*2*^ *= 0*.*753)*. There was also a significant interaction between the trial and the perturbation level (*F(4*.*299*, *64*.*485) = 0*.*931 with the Greenhouse-Geisser correction*, *p < 0*.*001*, *partial η*^*2*^ *= 0*.*058*). Using paired t-tests, we then examined the differences in changes in step length symmetry between time points for the two trials. There were significant differences over the entire adaptation period *(p = 0*.*0021*, *0*.*0016*, *0*.*0000*, *0*.*0007*, *0*.*0038*, *0*.*0054*, *0*.*0069*, *0*.*0122*, *0*.*0133*, *0*.*0163*, *0*.*0229*, *0*.*0055*, *0*.*0209*, *0*.*0124*, *respectively)*. During the post-adaptation period, we observed that the implicit VFD+SBT group deadapted much slower than the SBT-only group (Fig 3A). Also, two-way repeated measures ANOVA results showed a significant effect of trials (*F(1*,*15) = 8*.*086* with the Greenhouse-Geisser correction, *p<0*.*05*, η^2^ = 0.350) and significant interaction between trial and time on the rate of deadaptation (*F(7*.*49*,*112*.*356) = 3*.*550 with the Greenhouse-Geisser correction*, *p<0*.*001*, *η*^*2*^ *= 0*.*191*). This shows that the retention of aftereffects changed differently between the SBT-only and the implicit VFD+SBT trials during the post-adaptation period. Paired t-test results showed the differences in the group size of aftereffects over the entire post-adaptation period except for the first six minutes. *(p = 0*.*0052*, *0*.*0141*, *0*.*0101*, *0*.*0014*, *0*.*0053*, *0*.*0026*, *0*.*0052*, *0*.*0111*, *0*.*0027*, *0*.*0020*, *0*.*0022*, *respectively)*.

Fig 3B shows the results from the non-adapted group (11 subjects) who did not show visuomotor adaptation in response to the implicit VFD while walking on a split-belt treadmill. Statistical analysis using two–way repeated measures ANOVA on the step length symmetry revealed no statistically significant differences in step length symmetry changes over time in both the adaptation and the post-adaptation periods.

**Fig 4:**
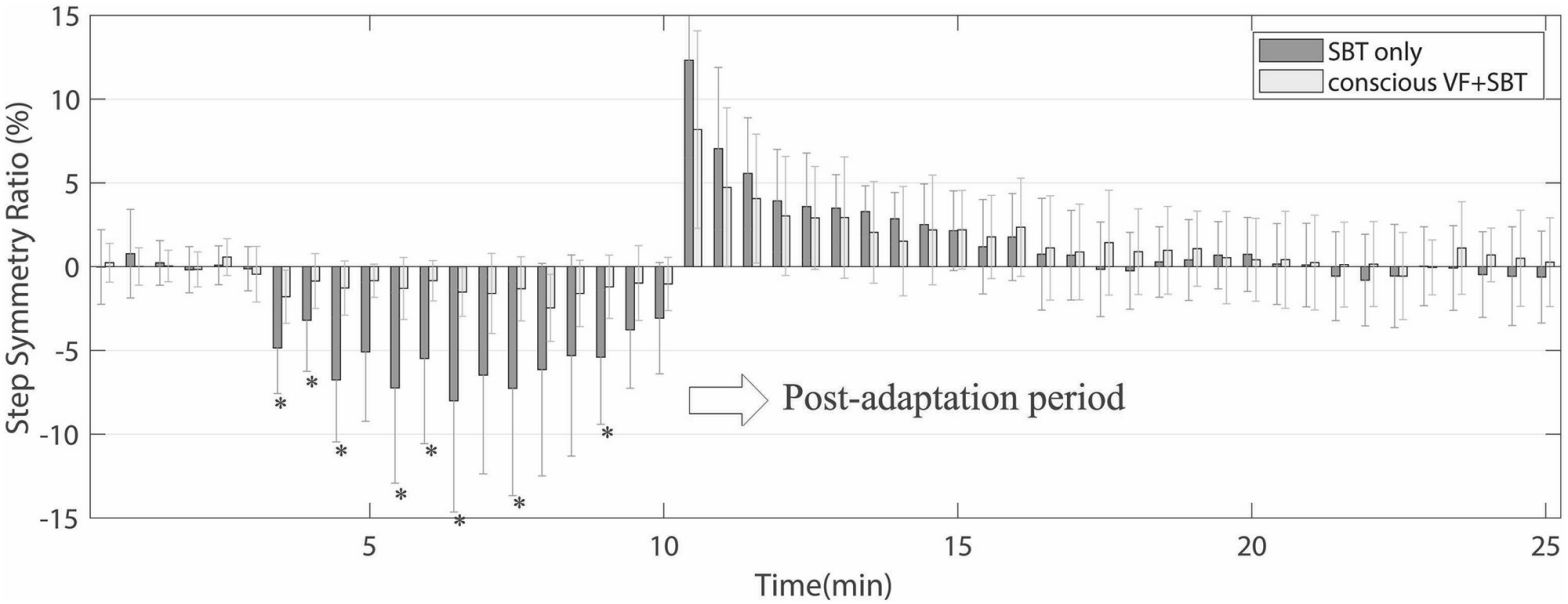
Changes in the mean step length symmetry averaged across 12 subjects during the conscious VF+SBT and the SBT-only conditions. The asterisks (*) were marked at time periods, where the induced step length symmetry values were significantly different between two conditions (p<0.05).

Fig 4 shows the group means of step length symmetry changes between the conscious VF+SBT trial versus the SBT-only trial. Only 12 subjects participated in the conscious VF+SBT trial, where they were instructed to match the veridical visual feedback bars during the adaptation period. We compared the difference in the retention of aftereffects of adapted symmetric gait patterns between the two conditions. The results of the two-way repeated measures ANOVA revealed a significant main effect of the trial on step length symmetry during the adaptation period (*F(1*,*11) = 5*.*695 with the Greenhouse-Geisser correction*, *p < 0*.*05*, *η*^*2*^ *= 0*.*341*), but no significant difference in the retention of aftereffects during the post-adaptation period. During the adaptation period, paired t-test results showed significant differences in changes in step length symmetry most of the time *(p = 0*.*0223*, *0*.*0134*, *0*.*0023*, *0*.*0244*, *0*.*0199*, *0*.*0279*, *0*.*0103*, *0*.*0333*, *respectively)*.

**Fig 5:**
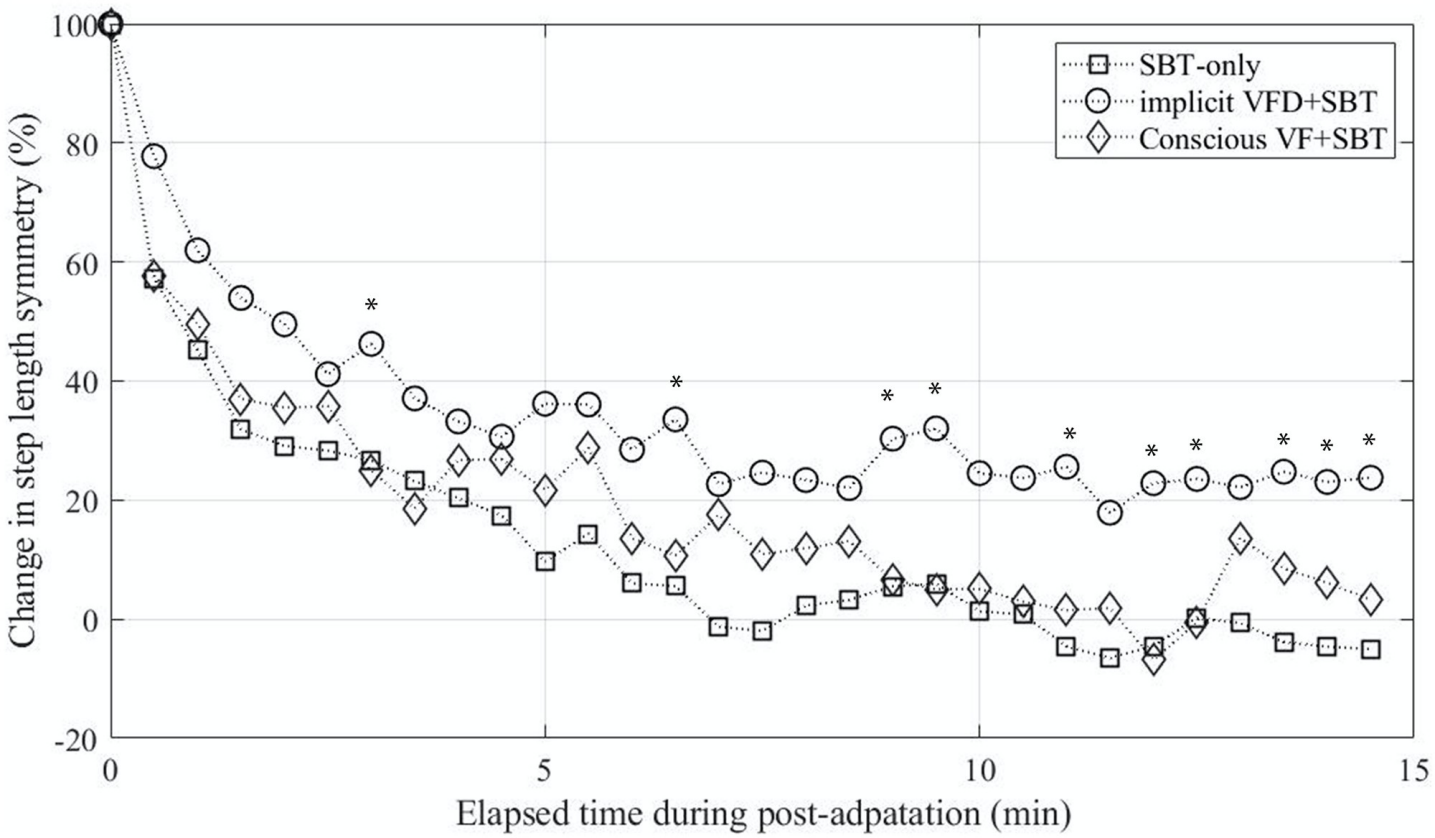
Change in step length symmetry during the post-adaptation period. Group means step length symmetry values (19 subjects for the SBT-only, 16 subjects for the implicit VFD+SBT, and 12 subjects for the conscious VF+SBT) were normalized by the initial values (100%). Displaying standard deviation and significant differences were ignored in this plot (refer to Figs 3A and 4).

Fig 5 shows the group means of retention (de-adaptation) rate changes during the post-adaptation period for the three trials. For computing the group means of the SBT-only trial, we did not include the data from the non-adapted group who did not show visuomotor adaptation in response to implicit VFD. However, if any subject in the non-adapted group participated in the conscious VF+SBT trial, we include their data in the SBT-only trial group mean for comparison purposes. Thus, this figure shows the group means values averaged across 12 subjects, 16 subjects, and 19 subjects for the conscious VF+SBT, implicit VFD+SBT, and SBT-only trials. Also, the step length symmetry data was normalized by an initial aftereffect value. Compared to the implicit VFD+SBT trial, the short-term retention of adapted step length symmetry was less pronounced in both the SBT-only and the conscious VF+SBT trials, indicating that the adaptation was stored less prominently. To investigate differences in the retention rate among the three experimental conditions in more detail, one-way repeated measures ANOVAs were used to examine the retention changes in the adapted symmetric gait pattern during the post-adaptation period. This test showed a significant main effect, and *post-hoc* analyses were then performed using the Bonferroni correction for pairwise comparisons among the three conditions (see marked time points in Fig 5).

## Discussions

In this study, we evaluated the potential benefits of concurrent gait training that combined a visual feedback distortion (VFD) paradigm with split-belt treadmill (SBT) walking in a single gait training by investigating the robustness of aftereffects of a learned gait symmetric pattern on a short-term basis. Understanding how gait adaptation and learning processes operate to make long-lasting changes in motor learning is crucial in gait rehabilitation. Our main finding was that there was an advantage of the implicit manipulation of visual feedback of gait symmetry in an unconscious manner for the longer-lasting effect of a newly learned gait pattern. However, not all subjects responded to the implicit VFD similarly. For the subjects who did not show visuomotor adaptation in response to the implicit VFD, the longer retention of aftereffects did not manifest.

### Effects of implicit visual feedback distortion on split-belt treadmill walking adaptation

In the implicit VFD+SBT trial, subjects walked on split-belts moving at different speeds while looking at the implicitly imposed distortion of visual feedback of their step length. The right step was initially shorter than the left one due to the asymmetric speed perturbation during the adaptation period. The right step length’s visual bar was only gradually distorted by being shorter than the actual step length. The subjects were given no instructions other than to walk comfortably while looking at the screen. We observed that subjects spontaneously adapted to the asymmetric visual information by producing longer right strides than those in response to the SBT-only trial. As shown in Fig 3A, this made gait asymmetry closer to the baseline during the adaptation period compared to the SBT-only trial. Significant differences were also found in the retention of aftereffects between the two conditions during the post-adaptation period. The results of this study demonstrated that SBT walking with the implicitly distorted visual feedback in an unconscious manner retained the acquired asymmetrical gait pattern longer than the SBT walking only, suggesting that sensory-prediction error unconsciously driven by the VFD influences retaining aftereffects longer. We found, however, that 11 subjects among our subjects did not show visuomotor adaptation in response to the implicit VFD (Fig 3B). Interestingly, there was no difference with the SBT-only trial in gait symmetry changes in both the adaptation period and the de-adaptation periods in this group. Although a further investigation should be conducted to ascertain why some subjects do not adapt in response to the VFD, from our previous VFD experimentation experience, visuomotor adaptation did not tend to occur when subjects seemed distracted during tests or often turned away from the screen. If a subject had not faithfully paid attention to the visual information displayed on the screen, the results would have been the same as the SBT-only trial, as shown in our results (Fig 3B). This study did not measure whether the subjects were constantly looking at the screen, and it was not easy to quantitively measure how much they engaged in the trials. Since the implicit VFD+SBT trial was based on an environment without the subjects’ intention during the performance, we realized the importance of motivating subjects to engage in a trial. When applying our proposed method to clinical applications in the future, more research should be conducted on characterizing the experimental environment in which subjects can more consistently produce visuomotor adaptation under unconscious conditions.

### Comparison of implicit visual distortion vs. conscious correction on retention of aftereffects

Gait adaptation appears to accelerate when training interventions involve subjects’ conscious (voluntary) correction in an explicit manner. Conscious repetition of a step asymmetry can cause a use-dependent bias toward aftereffects [24]. While encouraging voluntary (consciously) corrections during training is beneficial in quickly forming new motor patterns, it has been suggested that unconscious (automatic) processes involved in motor adaptation can lead to long-lasting retention effects or reduce the amount of forgetting over time compared to conscious (explicit) processes [8, 20, 25–27]. To understand this better, we compared the implicit visual feedback condition results to those from the conscious correction condition by assessing the rate of aftereffects from both conditions. During the conscious correction condition (conscious VF+SBT), subjects walked under the SBT-induced perturbation while consciously correcting their asymmetric gait patterns. To aid them in accomplishing this task, the veridical visual feedback displayed the bars representing the right and the left step lengths. Subjects were then instructed to match the height of the two bars on display with each step. As shown in Fig 4, as the subjects consciously corrected the asymmetric gait pattern induced by SBT walking, their step length symmetry stayed close to the baseline (toward symmetric gait). This indicates that the subjects had to produce longer right and left steps during the adaptation period. In other words, the subjects had to experience a larger magnitude of gait asymmetry than in the implicit VFD+SBT trial. Nonetheless, the aftereffects of the acquired asymmetric gait pattern still tended to decay quickly, and there was no significant difference in the retention rate compared to the SBT-only trial. When we looked at the deadaptation rate among the implicit VFD+SBT, conscious VF+SBT, and SBT-only groups, our results revealed that the aftereffects in the implicit VFD+SBT trial stayed significantly longer than in two other trials (Fig 5). In recent years, several studies by Wood et al. [24, 28] investigated asymmetric gait adaptation using a similar visual feedback distortion paradigm and showed that sensory-prediction error via distorted visual feedback did not improve storage of learning when subjects’ conscious movements were involved in a training task. Interestingly, although the experimental conditions were not entirely the same, our conscious trial results seem to be consistent with their results: the conscious VF+SBT trials where subjects consciously corrected their steps while matching the bars did not improve the retention rate compared to the SBT-only trial. This may suggest that the adapted motor behavior could be more prominently stored (at least on a short-term basis) when the visuomotor adaptation process via the implicit VFD is decoupled with consciously repetitive movements.

### Potential benefits of engaging the implicit (unconscious) motor learning process in gait training

Effective interventions need to facilitate a more efficient motor adaptation and ensure more persistent changes in newly learned motor patterns. Various researchers have suggested that the mechanism underlying the retention benefit of learning is the reliance on the unconscious (automatic) mode of a learning process that may accompany dual-task (e.g., distraction task), random practice, and visual perturbation [9, 19, 20]. The reason why implicit learning allows learned motor behaviors to remain longer has not yet been clarified. We speculate that the brain areas underlying implicit learning develop earlier in the period of growth and are, therefore, more effective at retaining motor learning [29]. When subjects were instructed to explicitly think about their movements, whether a given visual feedback was distorted or veridical, different brain systems could be engaged than those involved during implicit learning [18]. However, the precise role of the implicit process in the adaptation process needs to be further elucidated through future studies. Split-belt walking is known to alter gait patterns by employing both explicit and implicit processes [19]. During split-belt walking, however, subjects are more likely to perceive the speed difference of the split-belt, which may require a slight conscious effort and may reduce the ability to retain aftereffects longer. The SBT walking paradigm can be easily utilized to facilitate adaptation [30], whereas the implicit VFD paradigm within an unconscious (attention-independent) manner can have longer-lasting effects on a newly learned walking pattern [17, 22]. Indeed, the results of this study corroborate the effects of implicit VFD on the retention of symmetric gait patterns acquired with SBT walking. Our results demonstrated the potential advantages of combining different types of perturbation (implicit visual distortion and mechanical perturbation) in improving retention through an additive effect of each perturbation. Overall, our results support the idea that the gait changes spontaneously induced by visual distortion result in an augmented implicit learning mode, and its effect can still be sustainable when the visual distortion is implemented with split-belt treadmill walking.

### Limitations and further directions

This study evaluated the aftereffects (de-adaptation) rate in integrating implicit visual feedback distortion into SBT walking and conscious correction with SBT walking. However, our study is not without limitations. The current study focused only on the spatial aspects of walking, and the temporal elements were not explored. Second, regarding the limitations of this study’s experimental protocol, the post-adaptation period was only 15 minutes, and the speed of the slower belt was fixed at 2 mph (0.894 m/s). Certainly, the longer the period, the better understanding of the longer-term effect. If subjects were tested in a preferable walking speed environment, they might have shown a different amount of motor adaptation. Third, during SBT perturbation, only the speed of the right belt was increased regardless of the subjects’ leg dominance (preferential use of one leg over another). Gait asymmetric adaptation may vary slightly from individual to individual depending on leg dominance. Fourth, although the subjects were instructed to walk while continuously looking at the VFD, we could not quantitively measure how attentive they were to the instructions. Therefore, when a subject did not show visuomotor adaptation, we cannot confirm whether it was a tendency inherent in an individual or was just because the subject was not fully attentive during the trials. Future studies will be needed to answer these questions. Finally, in our study, there may be various independent experimental variables, such as whether there is visual feedback during SBT walking, whether distortion is applied to visual feedback, and whether explicit instructions are given to subjects. Future research should require a more rigorous and better design for an experimental protocol to explicitly demonstrate the effects of each independent variable involved in our experiments.

In conclusion, this study examined how combining two different forms of error-driven adaptation trials would affect storing a learned motor pattern (retention of a learned pattern). We found that SBT walking with the distorted visual feedback in an unconscious manner retained the acquired asymmetrical gait pattern longer than SBT walking only. Additional gait pattern changes made consciously during SBT walking showed little effect on increasing retention. A better rehabilitation program can be considered as one that enables longer retention of learned motor patterns than others. In this respect, our results suggest that integrating visual feedback distortion into split-belt treadmill rehabilitation could potentially enhance the outcome of gait therapy with longer retention by providing an implicit mode of learning. For better rehabilitation strategies, some form of visual feedback is often incorporated into training programs to enhance patients’ participation [31], and the method of using visual feedback distortion in an unconscious manner could be an effective rehabilitation program. However, further study should seek treatment strategies that can induce more consistent gait adaptation even under unconscious training conditions and determine the long-term functional effects of the visual feedback distortion paradigm on the overground transfer of an adapted step length symmetry. Further research quantifying the extent of interaction of explicit and implicit control of gait is also needed to fully understand the mechanisms underlying the rate of de-adaptation.

## Supporting information

S1 Dataset (XLSX)
